# Anti-VEGF drugs in the prevention of blindness

**Published:** 2014

**Authors:** David Yorston

**Affiliations:** Consultant Ophthalmologist: Tennent Institute of Ophthalmology, Gartnavel Hospital, Glasgow, UK. dbyorston@btinternet.com

**Figure:**
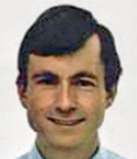
David Yorston

Disorders of the blood vessels in the retina are responsible for some of the most common causes of blindness in the world. These include:

- retinopathy of prematurity (an important cause of blindness in children in middle-income countries)
- diabetic retinopathy (the most common cause of blindness in the working-age population of industrialised countries)
- age-related macular degeneration (the third most common cause of blindness in the world).

All of these conditions are caused partly by over-production of a protein called vascular endothelial growth factor (VEGF). This protein was discovered in the 1980s and is important in the growth and development of blood vessels. VEGF production is increased by hypoxia (a lack of oxygen). So, if a tissue is not getting enough oxygen, it will produce more VEGF, which will stimulate the growth of additional blood vessels to provide more oxygen. This is beneficial in the heart muscle, or in a growing baby; however, in the eye it can be harmful.

The effects of VEGF may be summarised as:

- Increased permeability of existing blood vessels, causing them to leak.
- Growth of new blood vessels, which may bleed or leak fluid and proteins.

In the eye, both can lead to retinal damage.

Normal retinal capillaries (very fine blood vessels in the retina) are sealed thanks to tight junctions between the cells making up the capillary walls. This means that large molecules, such as proteins and lipids, cannot leak out of these retinal capillaries into the retina. In the presence of excessive VEGF, however, the capillaries start to leak and large molecules form exudates and escape into the retina, causing oedema in the surrounding tissues. If this affects the macula, then the central vision will be reduced. This is what causes diabetic macular oedema.

Excessive VEGF also causes the growth of new, abnormal retinal blood vessels and capillaries. These do not grow within the retina, where they might be useful.

Instead, the abnormal capillaries grow out from the retina onto the surface of the vitreous, where the vitreous touches the retina. This happens in proliferative diabetic retinopathy and retinopathy of prematurity. The new vessels are fragile and prone to tearing. When a new vessel is torn, it bleeds, causing vitreous haemorrhage. As the vitreous contracts, the new vessels pull on the retina, causing a traction retinal detachment. If the detachment includes the macula, vision will be impaired.

In the presence of excessive VEGF, new vessels can also grow out from the choroid (the layer immediately under the retina). These new vessels grow into the space between the retina and the choroid, usually just under the retinal pigment epithelium. The new vessels leak exudates formed of fluid or blood, causing oedema; eventually a fibrous scar is formed that destroys the photoreceptor cells at the centre of the macula. This is what happens in exudative (or ‘wet’) age-related macular degeneration (AMD).

Because of these very damaging effects in the eye, researchers have been working for years to find a way to block the activity of VEGF in the eye.

## Anti-VEGF drugs

VEGF has many beneficial effects in the rest of the body. Therefore, any anti-VEGF drug has to be given by a route that gives the maximum effect in the eye, but little or no effect elsewhere. In practice, this means it must be injected into the eye (intraocular injection). This carries a number of risks, and it is thought that about 1 in every 1,000 injections has a serious complication such as cataract, vitreous haemorrhage, retinal detachment, or infection. Each injection must be handled with appropriate sterilisation and aseptic technique (see page 47).

The two most widely used drugs at present are Lucentis (ranibizumab) and Avastin (bevacizumab). Both drugs are monoclonal antibodies that bind to all three forms of VEGF. They are very similar drugs (see page 48), but Lucentis is a smaller molecule and is believed to bind VEGF in the eye with greater affinity. Lucentis is intended purely for intraocular injection, and each vial can only be used for one patient. Avastin was intended to be given intravenously as an anti-cancer drug, and comes in vials of 100 mg.

As the dose required for intravitreal injection is only 2.5mg, one vial of 100 mg of Avastin can be used to treat 40 patients, provided that you have the necessary skills and facilities to prepare sterile injections for intraocular injection. In the United Kingdom, the British National Formulary gives a net price of £242* for a 100 mg vial of Avastin, (containing 40 doses). The British National Formulary gives a net price of £742* for a single 2.5 mg dose of Lucentis. This means that a single dose of Avastin can work out to be over 100 times cheaper than a single dose of Lucentis (provided that you have the skills and facilities, as stated above). [*Note: The actual prices of drugs vary considerably due to local purchasing arrangements, and may be very different from the net prices quoted above. These are taken from the British National Formulary, and only give an indication of the relative costs of the drugs.]

The most recently approved drug is aflibercept (also known as Eylea). This is an artificial protein that contains the VEGF receptor molecules that are normally attached to cell membranes. The VEGF binds to these receptors and is trapped and rendered harmless. Aflibercept seems to be as effective as Avastin and Lucentis, but may be given less frequently. In the British National Formulary, a single dose has a net price of £816.

### Side-effects

VEGF is important for the growth of new blood vessels. Although the dose of anti-VEGF needed to treat eye diseases is very small, it has been shown to reduce the level of VEGF in the bloodstream. This means that there is a theoretical risk that, in adults, anti-VEGF drugs may increase the risk of cardiovascular disease, including heart attacks and strokes. However, clinical trials have not shown conclusive evidence of an increased risk of cardiovascular disease in patients treated with anti-VEGF drugs compared to those given sham injections.

In babies and young children, VEGF is essential for the growth of blood vessels and normal growth and development of many other organs including the lungs, kidney and brain. This is one of the reasons why anti-VEGF drugs are absolutely contra-indicated in pregnancy. Women of childbearing age should have a pregnancy test prior to starting treatment, and must avoid pregnancy during treatment. Although Avastin has been used in the management of retinopathy of prematurity, its use is controversial, because of the potential for serious adverse effects when used in young babies (see panel on page 46).

In practice, the major adverse effects of these drugs appear to be associated with the intraocular injection rather than the active drug.

### Treatment regimes in adults

Regardless of the treatment indication, there are essentially two regimes for administering anti-VEGF drugs: **continuous** and **intermittent/as required** (or *pro re nata*, **PRN** for short).

Most of the initial trials were done as a **continuous** regime, with regular monthly injections over the course of 2 years, i.e. patients would have 24 injections in total. These trials showed that treatment delivered in this way was effective, but it is also expensive and inconvenient for both the patient and the health care provider.

A number of other trials have examined **PRN** regimes. These are all fairly similar and consist of three injections given over 3 months, followed by review. At this point the patient may:

- be much better, in which case no additional treatment is needed
- have no improvement at all, in which case further treatment is futile
- have some improvement, which would justify further injections.

Even among those patients who do very well, and do not require more than three injections initially, many will relapse and require further injections in the future.

This means they must be regularly reviewed in the clinic, i.e. every 1–2 months, which involves testing of visual acuity and/or retinal thickness. If patients’ visual acuity is lower by one or more lines, or if they develop worsening macular oedema, they need a further injection of anti-VEGF.

Trials of this dosing regime for AMD and diabetic macular oedema have shown that an average of seven injections are required in the first year of treatment and that outcomes are as good as for regular monthly injections. The trials used as their indication for re-treatment with anti-VEGF either a reduction in visual acuity or an increase in retinal thickness (measured using optical coherence tomography [OCT]); both were assessed at each visit.

While visual acuity is easily measured, retinal thickness can only be measured accurately by OCT. These machines are costly and will only be available in major cities in low- and middle income countries. We don't yet know how effective PRN treatment might be when retreatment is guided by visual acuity alone, as both visual acuity and retinal thickness were measured at each visit in these trials.

Although intermittent regimes reduce the number of injections, patients still have to be reviewed every 1–2 months, which leads to very busy clinics; this is also a significant burden for the patients and for their families.

## Which diseases can be treated?

### Age-related macular degeneration

Lucentis, Avastin and Eyelea are equally effective in exudative AMD. There appears to be little difference between monthly and PRN dosing. The average (mean) improvement in vision is about 1–2 lines, and about one-third of patients will improve by three or more lines. With a PRN regime, an average of seven injections will be required in the first year. Most of these trials excluded eyes with a vision of less than 6/96 (4/60), and treatment is unlikely to be effective in advanced exudative AMD with sub-macular scarring or a vision of ‘counting fingers’ or ‘hand movement’. Unfortunately, exudative AMD often co-exists with atrophic (‘dry’) AMD, and the anti-VEGF drugs only treat the exudative component. With longer follow-up (over 2 years), atrophic AMD may cause a gradual loss of vision despite effective anti-VEGF treatment.

### Diabetic macular oedema

Once again, there seems to be little difference between the different anti-VEGF drugs. All three are effective at treating diabetic macular oedema, and the average improvement in vision is about 1.5 lines. Roughly 25% of patients will have their visual acuity improve by three or more lines and 50% by two or more lines. An average of seven injections will be required in the first year of treatment with a PRN regime.

Not all patients with diabetic macular oedema need to be treated with anti-VEGF. Laser treatment still has an important role: macular oedema which does not involve the fovea is best treated with laser. These patients will normally have good vision, and the laser will help to preserve it. Moreover, laser is usually effective with a single treatment, which is much easier for the patient than repeated monthly injections.

If new vessels are present, they should be given pan-retinal laser treatment first, before any macular oedema is treated using anti-VEGF. This is because anti-VEGF makes the new vessels regress very quickly. As the treated vessels become fibrotic, they contract, which can cause a retinal detachment.

### Retinal vein occlusion

There is good evidence from clinical trials that all three anti-VEGF drugs will reduce the risk of loss of vision following central retinal vein occlusion. About 50% of patients will gain three or more lines, with a mean improvement of about two lines. There is also a reduced risk of rubeosis and secondary glaucoma with anti-VEGF treatment.

Lucentis has been shown to improve outcomes after branch retinal vein occlusion as well. However, as many of these patients will improve spontaneously, this evidence is not quite as strong.

In summary, anti-VEGF drugs are probably the most significant advance in ophthalmology in the last decade. They have enabled us to treat what were previously untreatable conditions. They are not a perfect solution, however.

- They do not cure the underlying problem, so repeated treatment is necessary and most patients will require a lifetime of regular monitoring.
- The drugs are expensive, and even high-income countries have struggled with the costs and logistics of delivering thousands of intraocular injections every year. Although anti-VEGF drugs are the most effective treatment for many retinal diseases, the visual improvement is modest, averaging about two lines of vision. Relatively few patients will regain normal vision.
- Patients who present late, with very advanced disease and a visual acuity of less than 3/60, may not benefit from treatment.
- Most PRN treatment regimes rely on OCT imaging, which is rarely available in low and middle income countries. We have little information on the use of anti-VEGF in this setting, and we cannot be sure that the good results achieved in Europe and North America will be replicated in Africa, India, or China.

Despite these reservations, anti-VEGF drugs are going to play an increasing role in the prevention of blindness worldwide. As the global population ages, and becomes more overweight, both AMD and diabetic retinopathy will become more common. The drugs will become cheaper, and we may find better ways of monitoring treatment so that expensive OCT is no longer essential.

